# Easy Identification of *Leishmania (Leishmania) amazonensis* and *Leishmania (Viannia) braziliensis* Species by Using Fourier-Transform Infrared Spectroscopy and Machine Learning Algorithms

**DOI:** 10.1021/acsomega.5c05522

**Published:** 2025-10-29

**Authors:** Vilma A. S. Oliveira, Vitoria S. Fernandes, Fernanda Silva, Thiago Franca, Camila Calvani, Bruno Marangoni, Carla Arruda, Cicero Cena

**Affiliations:** † UFMS − Universidade Federal de Mato Grosso Do Sul, Laboratório de Parasitologia Humana, Instituto de Biociências, Av. Costa E Silva S/N, Campo Grande, MS 79070-900, Brazil; ‡ Laboratório de Fotodiagnóstico, Instituto de Física, 54534 UFMS − Universidade Federal de Mato Grosso Do Sul, Av. Costa E Silva S/N, Campo Grande, MS 79070-900, Brazil

## Abstract

Leishmaniasis is a neglected tropical disease requiring accurate species identification to ensure proper clinical management and epidemiological surveillance. Accurate species and strain identification depends on molecular and biochemical tools. While these conventional techniques are effective, they are often costly, time-consuming, and inaccessible in low-resource settings. In this study, we evaluated the potential of Fourier-transform infrared (FTIR) spectroscopy, combined with machine learning algorithms, for the discrimination of *Leishmania amazonensis* and *Leishmania braziliensis* species in liquid cultures. FTIR spectra were acquired from 80 culture samples and preprocessed using standard normal variate (SNV) correction and Fast Fourier Transform (FFT) filtering. Principal Component Analysis (PCA) revealed clear species-specific clustering driven by spectral differences in protein, lipid, and nucleic acid vibrational bands. Support Vector Machine (SVM) models were trained using PCA scores, achieving over 90% accuracy in all tested configurations. The best model, using a linear kernel and the first three principal components, reached 100% accuracy, sensitivity, and specificity in external validation. Our findings demonstrate that FTIR spectroscopy, in combination with SVM, offers a rapid, low-cost, and scalable strategy for the screening and classification of *Leishmania* species, with promising applications for field and clinical diagnostics.

## Graphical abstract

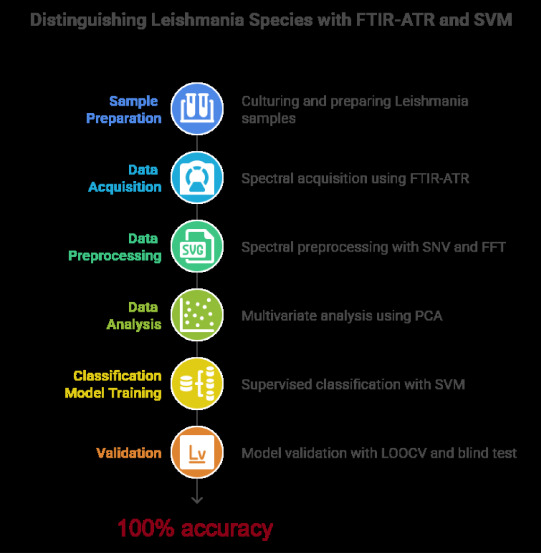

## 1 Introduction

Leishmaniasis encompasses a group of neglected tropical diseases (NTDs) that affect populations across all five continents.^1^ Each year, an estimated 1.3 million new cases and 20,000 to 30,000 deaths occur globally. In the Americas, the visceral, cutaneous, and mucocutaneous clinical forms pose a significant public health threat. In Brazil alone, health authorities reported 1,648 cases in 2022, including 105 in the Centro-Oeste region.^2^

Treatment, management, and control of leishmaniasis are constant challenges, mainly due to insufficient investment in novel therapies, preventive strategies, diagnostic tools, and even accurate identification of species and strains. These limitations hinder disease control efforts and disproportionately affect vulnerable populations worldwide.^3^ Precise identification of *Leishmania* species remains critical for optimizing clinical interventions; however, research in this area remains underdeveloped.^4^

Accurate species and strain identification depends on molecular and biochemical tools such as high-resolution melting (HRM) real-time polymerase chain reaction (PCR), DNA sequencing, PCR-restriction fragment length polymorphism (PCR-RFLP), and isoenzyme analysis. PCR-HRM demonstrates 96.4% sensitivity in biopsy samples^5^ and offers rapid, adaptable analysis. DNA sequencing provides high-resolution identification (≥99% accuracy)^4^ but requires specialized infrastructure.^6^ PCR-RFLP and isoenzyme analysis remain valuable for species differentiation,^6,7^ although isoenzyme-based techniques are labor-intensive and vulnerable to contamination due to parasite culturing.^7^ These methods enable the differentiation of key pathogenic species, such as *L. braziliensis* (mucocutaneous) and *L. donovani* (visceral), yet vary widely in cost, accuracy, and operational feasibility.

Despite their efficacy, the global application of these techniques encounters several barriers. These include the need for advanced infrastructure—limiting their use in resource-limited endemic regions, genetic variability among Leishmania species (e.g., kDNA variants), and sample degradation. Furthermore, the absence of standardized protocols for molecular markers (such as hsp70) and the presence of coinfections (e.g., *L. amazonensis* with *L. infantum*) complicate diagnosis. Traditional methods often rely on parasite culturing, which delays treatment and introduces additional logistical challenges.^5–7^

Accurate species identification plays a critical role in guiding targeted therapies, as each clinical form requires distinct treatment approaches (e.g., antimonials for visceral leishmaniasis vs amphotericin B for mucocutaneous leishmaniasis). Effective species-level identification also enhances epidemiological surveillance by enabling precise mapping of reservoirs (e.g., dogs) and vectors (e.g., sand flies).^8^ Misdiagnosis not only delays treatment but also increases the risk of drug resistance. Integrating diagnostic data from human, animal, and environmental sources through a *One Health* framework is essential for breaking zoonotic transmission cycles and mitigating the global burden of leishmaniasis.^9^

Challenges in *Leishmania* species identification reflect broader limitations in the diagnosis of other infectious diseases. In response, researchers have explored optical spectroscopy techniques as promising, low-cost alternatives.^10,11^ These techniques analyze spectral signatures from biological samples with minimal processing, offering a practical solution for low-resource settings.

Among these alternatives, Fourier-transform infrared (FTIR) spectroscopy has shown strong potential for microbial identification, combining low cost with easy implementation—two critical factors for public health and neglected disease control.^12–14^ FTIR probes the vibrational modes of molecular bonds in the sample matrix. Subtle changes in concentration, molecular composition, conformation, and environmental conditions (e.g., pH) influence spectral signatures and allow group classification through proper data analysis. Integrating FTIR with machine learning enables enhanced classification, model validation, and interpretability of spectral data.^12–14^

However, FTIR-based classification also faces limitations. Spectral features often reflect dominant molecular concentrations, which may obscure signals from diagnostically relevant but low-abundance molecules.^15^ In cultured samples, high promastigote concentrations do not guarantee informative spectra, as the surrounding matrix includes numerous nonspecific biomolecules. Differences between species primarily arise at the molecular level within the promastigotes themselves.^16^ Standard FTIR spectrometers often lack the sensitivity to resolve these subtle differences.

To mitigate these issues, many studies analyze dried samples,^12,13,17,18^ which offer improved spectral resolution but introduce practical challenges, including longer preparation times, strict control of temperature and humidity, sample heterogeneity, and the need for spatial measurements across multiple regions.

This study aims to discriminate *Leishmania* species obtained from axenic cultivation of reference strains using FTIR spectroscopy in liquid sample form. We seek to identify key spectral features contributing to group classification and to develop a robust, reproducible protocol for simple and scalable sample analysis.

## 2 Materials and Methods

### 2.1 Axenic Cultivation of *Leishmania* sp. Promastigotes

The samples that were subjected to FTIR analysis were reference strains that had already been properly identified and characterized using conventional methods:^19^ *Leishmania (Leishmania) amazonensis* (IFLA/BR/1967/PH8 and MHOM/BR/2022/LT 013_22 strains) and *Leishmania (Viannia) braziliensis* (MHOM/BR/75/M2904 strain). Parasites were maintained as promastigote forms at the Human Parasitology Laboratory/INBIO/UFMS in Schneider’s Insect Medium (SIM) (Sigma-Aldrich, SP/Brazil), supplemented with 20% fetal bovine serum (Sigma-Aldrich, SP/Brazil), 10,000 U/mL of penicillin, and 10 mg/mL of streptomycin (Sigma-Aldrich, SP/Brazil).^20^

Cultures were expanded in the stationary growth phase and analyzed on the fifth day of cultivation. To prepare the samples, both cultures were centrifuged at 3,500 rpm for 10 min, after which the supernatant (culture medium) was discarded. Subsequently, the parasite concentrate was diluted in 500 μL of medium and characterized by infrared spectroscopy.^20^

We divided the samples into two groups—*L. amazonensis* and *L. braziliensis* MHOM/BR/75/M2904)—with 40 samples in each group. The *L. amazonensis* group included two distinct strains: 20 samples from strain MHOM/BR/2022/LT 013_22 and 20 from strain IFLA/BR/1967/PH8. To increase intragroup variability and avoid bias associated with batch-specific effects, we performed independent culture replications across different weeks. Consequently, the samples were not derived from a single culture or measured under identical conditions on the same day. This approach aimed to capture natural variability within the group and improve the robustness and generalizability of the spectral classification.

### 2.2 Infrared Spectra Data Acquisition

We acquired the infrared spectra of each sample using an Agilent Cary 630 Fourier Transform Infrared (FTIR) spectrometer equipped with a solid attenuated total reflectance (ATR) crystal accessory. For each measurement, 20 μL of the liquid culture sample was directly deposited onto the ATR surface at room temperature. To account for bulk water absorption, background spectra were obtained using deionized water on the same crystal. Spectral acquisition was performed in the mid-infrared (MIR) region (1800–900 cm^–1^), using a resolution of 8 cm^–1^ and averaging 16 scans per sample.

In our configuration, each spectrum was acquired within approximately 30–40 s. In contrast, the preparation of dried samples typically requires around 30 min to allow for complete evaporation under controlled temperature and humidity conditions—excluding additional time needed to mitigate potential drying artifacts and spectral heterogeneity. Therefore, beyond minimizing sample manipulation, the use of liquid samples significantly improves the speed and practicality of the method, making it particularly suitable for routine and high-throughput applications.

### 2.3 Data Analysis and Prediction Model Validation

#### 2.3.1 Data Preprocessing

We performed all data analysis, predictive modeling, and figure generation using the Scikit-Learn library (version 1.3.0) in the Python programming language (version 3.11.5).^21^ Initially, the raw FTIR spectra underwent a preprocessing step. We applied a modified standard normal variate (SNV) normalization, using the absorbance at 1800 cm^–1^ as the reference baseline. Specifically, each spectrum *x = [x*_1_,*x*_2_, *..., x*_*n*_] was baseline-adjusted by subtracting the absorbance value at 1800 cm^–1^ and then standardized according to

$$x_{i}^{\prime }=\frac{\left(x_{i}-x_{1800}\right)-\mu }{\sigma }$$

where *x*_1800_ is the absorbance at 1800 cm^–1^, μ is the mean of the baseline-adjusted spectrum, and σ its standard deviation. This approach ensures that all spectra are centered relative to a consistent reference point (1800 cm^–1^) and scaled to unit variance before FFT filtering. This normalization technique minimizes baseline variability and compensates for multiplicative scatter effects across samples, improving spectral comparability.^22^

However, we observed that the raw spectra exhibited low signal values and the presence of interference fringes, high-frequency oscillations resulting from optical path differences in the ATR setup. These fringes introduce frequency-dependent artifacts into the spectra, potentially biasing group classification. To address this, we applied a Fast Fourier Transform (FFT)-based filter to smooth the spectra and suppress high-frequency noise. The FFT filter effectively removes periodic noise components while preserving the relevant spectral features, thereby enhancing signal quality and the accuracy of subsequent multivariate analyses.^23,24^

#### 2.3.2 Principal Component Analysis—Group Clustering

We subjected the preprocessed FTIR-SNV-FFT data to multivariate statistical analysis using Principal Component Analysis (PCA) to enhance and highlight sample similarities and differences based on the main spectral features associated with molecular vibrational modes.^25^ PCA reduces the dimensionality of the data set while preserving the variance, enabling visualization of intrinsic clustering patterns and discrimination among sample groups based on their chemical composition.

Prior to interpretation, a one-time Hotelling’s T^2^ test was applied to the PCA score matrix to identify and remove multivariate outliers.^26^ This test quantifies the distance of each observation from the multivariate center within the PCA-reduced space, taking into account the covariance structure of the data. Observations with T^2^ values exceeding the confidence threshold were flagged as outliers and excluded to ensure robust pattern recognition and prevent undue influence on group separation.

In this data set, PCA effectively captured the dominant sources of spectral variation and separated samples according to strain- or species-specific biochemical signatures. The PCA score plots revealed group clustering trends, while the corresponding loading plots identified the wavenumbers (i.e., vibrational bands) that most contributed to intergroup separation. These loadings provided insight into the molecular components underlying the classification, supporting the interpretation of biochemical differences between Leishmania species or strains.

#### 2.3.3 Prediction Model—Support Vector Machine

Finally, we employed a Support Vector Machine (SVM) algorithm to construct a predictive classification model using the PCA scores as input features.^27^ SVM is particularly well-suited for this type of analysis, as it constructs an optimal hyperplane that maximizes the margin between different sample groups in the multivariate space defined by the principal components. By utilizing a defined number of PCs, SVM can capture complex nonlinear relationships when appropriate kernel functions (e.g., linear, polynomial, radial basis function) are applied.

We optimized the model by systematically adjusting key hyperparameters—such as the regularization parameter (C), kernel type, and kernel-specific parameters—as well as by varying the number of principal components included in the model. To identify the optimal number of PCs, we evaluated classification accuracy in both training and validation data sets. We selected the configuration that achieved the highest overall accuracy while minimizing the discrepancy between training and validation performance, thereby reducing the risk of overfitting and improving model generalizability.^23^

Once trained and validated, the final SVM model can be exported and applied to external data sets for automated classification, eliminating the need for reaccessing the original training data and enabling practical deployment in diagnostic workflows.

#### 2.3.4 Validation Tests

The predictive accuracy of the model was estimated using the Leave-One-Out Cross-Validation (LOOCV) approach during the training phase.^28^ Model training was performed using 70% of the total sample set. In LOOCV, each sample is sequentially excluded from the training set and used as an independent test case, while the remaining samples are used to train the model. This process is repeated iteratively until every sample has been used once as the validation set. The overall accuracy is calculated as the average classification performance across all iterations.

After identifying the optimal model conditions, the remaining 30% of the data set—comprising samples not previously used during training—was employed as an independent test set. A confusion matrix was then constructed to assess the model’s robustness by quantifying correct and incorrect classifications, as well as deriving key performance metrics such as sensitivity and specificity. Figure 1 shows the pipeline of the experimental procedure.

**Figure 1:**
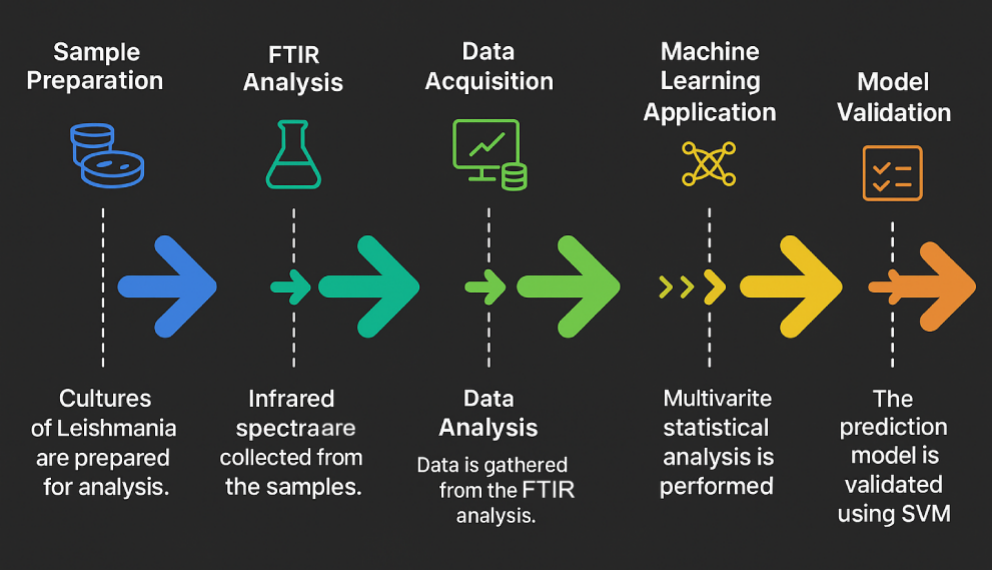
Flowchart of the experimental procedure summarizing the steps and activities until the achievement of a model for *Leishmania* species classification.

## 3 Results and Discussion

Figure 2a,b displays the average FTIR-SNV spectra of *Leishmania amazonensis* and *Leishmania braziliensis*, both before and after application of a Fast Fourier Transform (FFT) filter. The raw spectra exhibit characteristic interference patterns—periodic oscillations superimposed along the spectral range—which are likely associated with the nature of the liquid-drop samples and the single-reflection ATR (Attenuated Total Reflectance) configuration used during data acquisition. These spectral artifacts are characterized by an overall reduction in signal values, making the interference pattern more evident. Such effects likely arise from a combination of experimental conditions and optical factors (e.g., scattering, reflection losses, or alignment issues).

**Figure 2:**
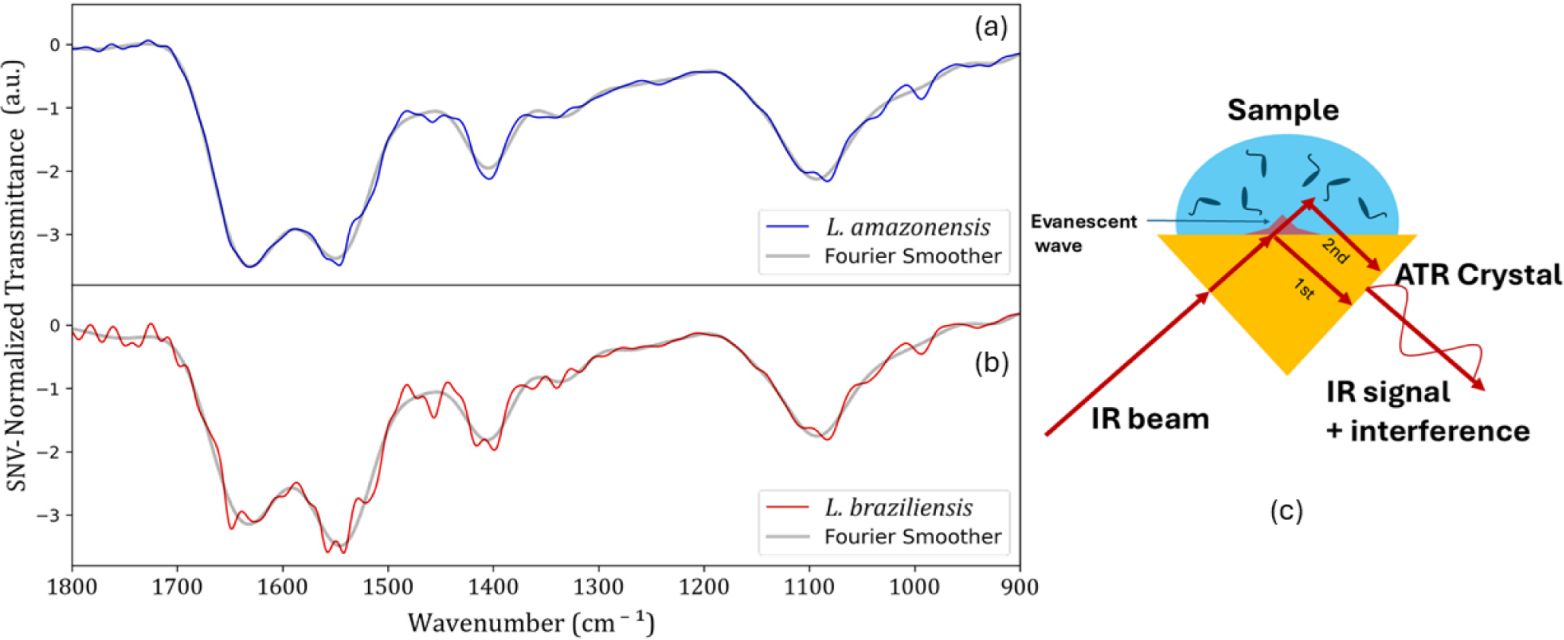
FTIR-SNV average spectra for: (a) *L. amazonensis* (blue trace) and (b) *L. braziliensis* (red trace) culture. (c) A diagram scheme to explain the origin of the interference pattern in the experiment. SNV-Normalized Transmittance values represent rescaled spectral intensities (min–max normalization), where 0 corresponds to the lowest relative intensity and 1 to the highest, not absolute physical transmittance.

One contributing factor to the transmittance below 10% is the limited concentration of biological material in the sample. When the concentration of cells is insufficient, fewer biomolecular constituents interact with the infrared radiation, resulting in lower absorbance values and faint or indistinct bands. To address this, increasing the cell density and minimizing the presence of residual culture medium, such as SIM, are important steps to enhance the quality of the spectra, such as improving the number of scans. However, additional procedures can complicate the methodology and become time-consuming. To maintain the goal of a fast and simple approach, we chose to use the samples as obtained to evaluate the robustness of the data treatment.

The interference fringes observed in the spectra are indicative of thin-film interference effects, which are common in ATR measurements involving nonuniform or thin liquid films.^29^ These fringes originate from multiple internal reflections occurring at the interfaces between the ATR crystal, the sample, and the surrounding air. When the sample behaves like a semitransparent thin film, constructive and destructive interference between the reflected IR beams generates periodic modulations in the spectrum. Furthermore, irregular sample thickness or inhomogeneous distribution, such as that caused by the droplet curvature or biofilm-like structures, leads to variations in optical path length, reinforcing the formation of such interference patterns. Figure 2c illustrates a didactic scheme describing such phenomena.

To minimize the impact of interference fringes and improve spectral quality, postacquisition data processing, such as baseline correction or the application of FFT filters, can be employed to suppress residual fringe artifacts and enhance the visibility of relevant spectral features. In this study, we applied an FFT filter to remove the periodic oscillations from the data set. Figure 2b, in particular, exhibits a more prominent effect of interference fringes, which could introduce bias in group classification during data analysis. The result after FFT filtering can also be identified in the figure (gray trace). This highlights the importance of a careful inspection of raw data before conducting multivariate analysis.

Figure 3 displays the FTIR-SNV-FFT average spectra for the *L. braziliensis* and *L. amazonensis* groups. In Figure 3a, the shaded regions represent the standard deviation within each group. The 1440–900 cm^–1^ range shows more spectral variability for both groups, indicating greater signal dispersion. In particular, the *L. braziliensis* group shows higher deviation in the entire spectrum when compared to the *L. amazonensis* group.

**Figure 3:**
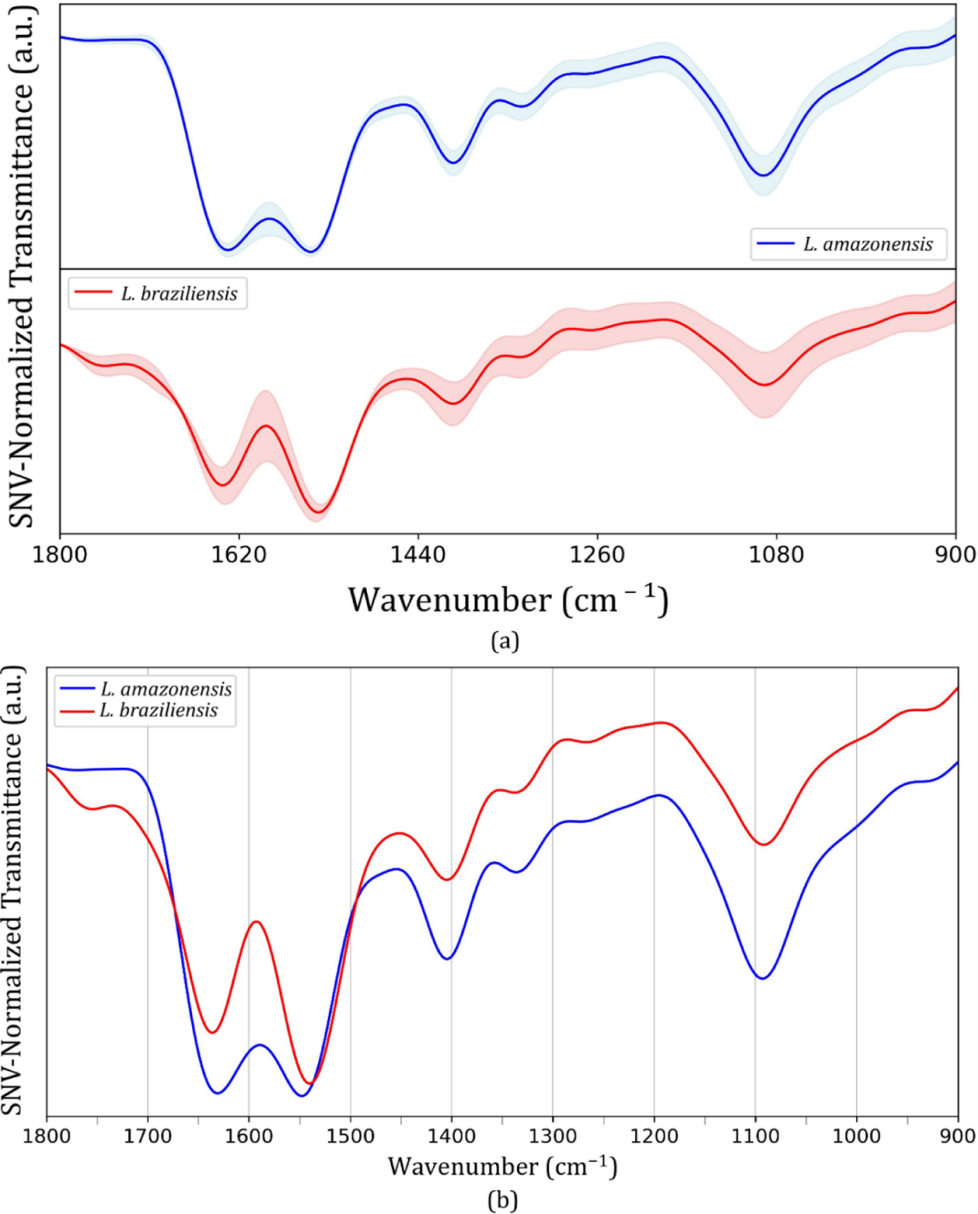
FTIR-SNV-FFT average spectra for *L. amazonensis* (blue trace) and *L. braziliensis* (red trace) cultures in Schneideŕs Insect medium. (a) shows the average spectra and the respective standard deviation for each group and (b) exhibits the superimposed average spectra for each group for comparison.

Figure 3b shows the superimposed FTIR-SNV-FFT average spectra for the *L. braziliensis* (red trace) and *L. amazonensis* (blue trace) groups. From 1500 to 900 cm^–1^, both groups exhibit a similar number of vibrational modes, with comparable spectral patterns and relative band intensities. However, notable differences emerge in the 1800 to 1500 cm^–1^ region. A minor band around 1750 cm^–1^ is observed exclusively in the *L. braziliensis* group. Additionally, the major bands centered at approximately 1650 and 1550 cm^–1^ display markedly different relative intensities between the two groups, suggesting distinct contributions for the vibrational modes emerging from the biomolecular matrix, which can be related to compositional and/or conformation changes.

The FTIR spectral analysis of *Leishmania* promastigotes cultured in SIM revealed distinct vibrational bands corresponding to molecular components derived from both the parasites and the culture medium. The prominent bands at ∼1750 cm^–1^ and ∼1650 cm^–1^ are attributed to the C=O stretching vibrations of amide I, primarily originating from protein constituents present in the SIM. The band at ∼1550 cm^–1^ corresponds to the N–H bending vibration of amide II, characteristic of the protein content of *Leishmania* cells. The absorption at ∼1400 cm^–1^ is consistent with symmetric stretching of CH_3_ groups from lipids, as well as contributions from the C=O stretching of carboxylate groups (COO^–^) and CH_2_ bending of lipid chains, reflecting the mixed biochemical origin from both the parasites and residual medium components. The band observed at ∼1350 cm^–1^ is indicative of in-plane bending vibrations of CH_3_ groups, likely arising from methyl-bearing biomolecules such as phospholipids or branched-chain amino acids. A band at ∼1280 cm^–1^ is assigned to overlapping amide III vibrations from protein backbones and P=O stretching modes from nucleic acids (DNA and RNA) of the parasite cells. Additionally, the symmetric stretching of PO_2_^–^ groups, also observed in this region, can be attributed to the phosphodiester backbone of nucleic acids from *Leishmania*, although potential contributions from phosphate-containing polysaccharides in Schneideŕs Insect medium cannot be excluded.^30,31^

Figure 4 presents principal component analysis (PCA) results derived from FTIR-SNV-FFT spectra of *L. amazonensis* (blue circle symbols) and *L. braziliensis* (red diamond symbols) groups. On the left, the scores plot (PC1 vs PC2) exhibits a clear separation between the two species, with PC1 accounting for 98.1% of the total variance and PC2 contributing 1.1%. This separation suggests strong biochemical differences in the spectral profiles of the two *Leishmania* species, most of which are captured by PC1, as expected, once we observed in the FTIR average spectra (Figure 3b) visible differences in the 1700–1500 cm^–1^ range.

**Figure 4:**
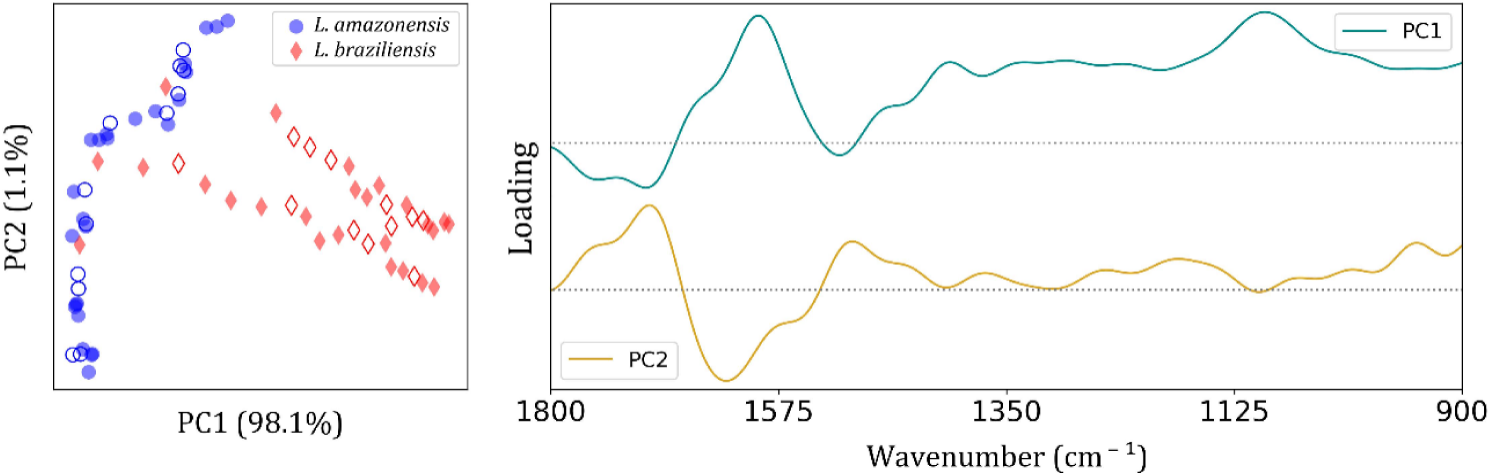
Principal Component Analysis (PCA) of FTIR spectra from *Leishmania amazonensis* and *Leishmania braziliensis* promastigotes culture in Schneideŕs Insect medium. (Left) PCA score plot showing clear separation between *L. amazonensis* (blue circles) and *L. braziliensis* (red diamonds). Filled symbols represent samples used for model training, while hollow symbols correspond to test samples reserved for model validation. (Right) PCA loading plots for PC1 and PC2, indicating the spectral regions contributing most to the variance. The dashed horizontal line denotes the zero-loading baseline for reference.

The corresponding loading plots (Figure 4 on the right) provide insight into the spectral regions responsible for the observed variance. For PC1, major contributions are observed around 1650 cm^–1^, corresponding to C=O stretching vibrations of amide I from proteins, and at ∼1550 cm^–1^, related to N–H bending vibrations of amide II, likely reflecting species-specific protein composition. Additional contributions appear near 1400 cm^–1^ (symmetric CH_3_ stretching and COO^–^ stretching), and around 1240–1280 cm^–1^, where overlapping amide III vibrations and *p* = O stretching from nucleic acids are found. The region between 1100 and 1000 cm^–1^, where symmetric stretching of PO_2_^–^ groups from nucleic acids and carbohydrates occurs, also contributes significantly to PC1.

PC2 captures smaller variance, with notable features at ∼1550 and ∼1400 cm^–1^, suggesting it may reflect subtler variations in protein or lipid composition rather than species-level differentiation. Overall, the PCA analysis indicates that spectral differences primarily related to proteins (amide I and II), lipids (CH_2_, CH_3_), and nucleic acid components (P=O and PO_2_^–^) drive the discrimination between *L. amazonensis* and *L. braziliensis* in the FTIR data set.

We acknowledge that some spectral bands, such as amide I (∼1650 cm^–1^) and amide II (∼1550 cm^–1^), may include contributions from both parasite and residual medium proteins. Nonetheless, as the same medium was used for all groups, these background contributions remained constant across samples. Therefore, the observed discrimination is attributable to species-specific biomolecular signatures rather than medium artifacts.

Figure 5 presents the overall accuracy of SVM-based classification models (left column) using different kernel functions (Linear, Quadratic, and Cubic) and varying the regularization parameter C (1, 10, and 100). For each configuration, the number of principal components (PCs) was incrementally increased, and overall accuracy was monitored. To reduce the risk of overfitting, model evaluation was halted at the first local maximum in accuracy. All tested configurations yielded accuracy above 90%.

**Figure 5:**
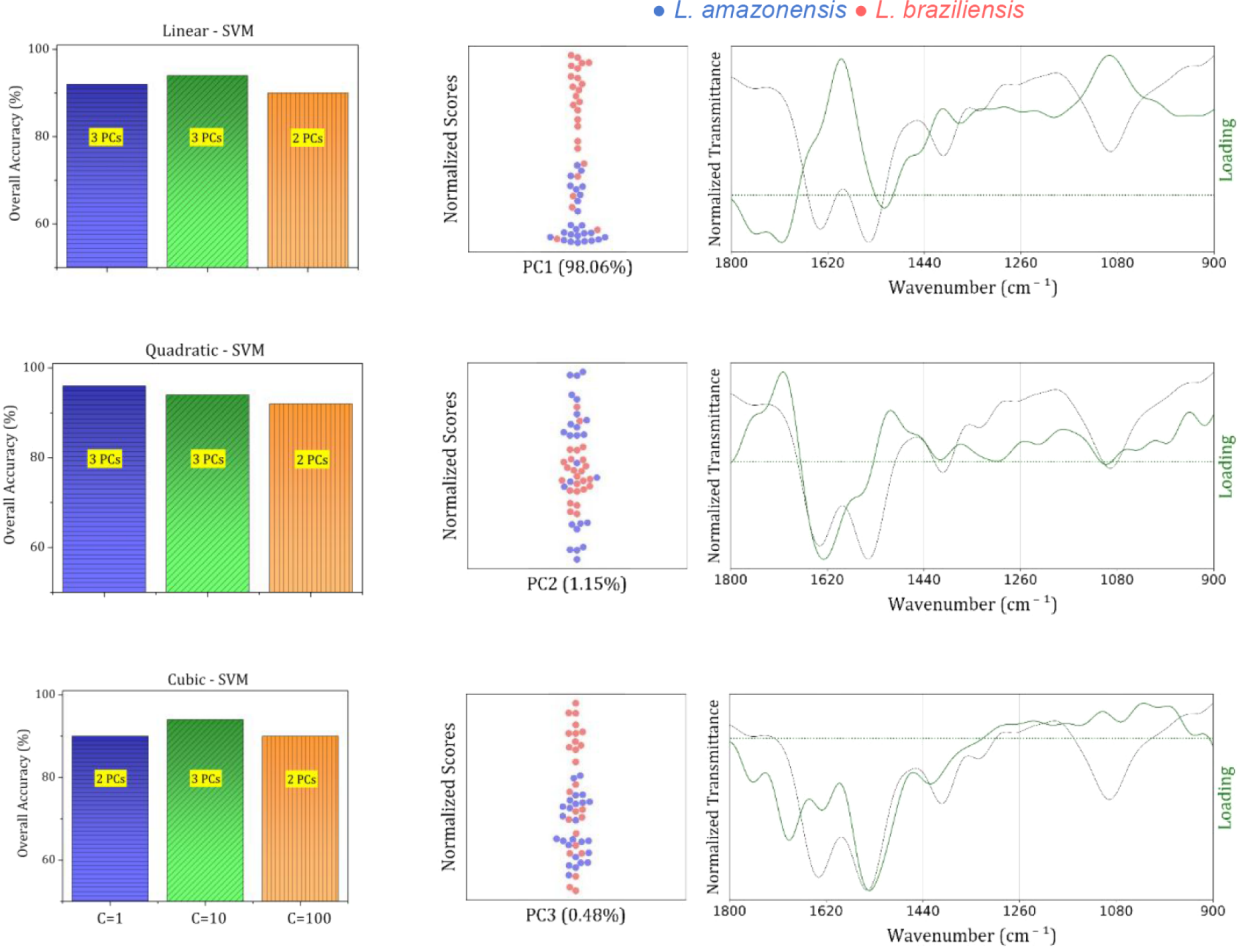
Performance of SVM classification models using line, quadratic, and cubic (top to bottom, left column) kernels for the discrimination of *Leishmania* species based on PCA data from FTIR-SNV-FFT spectral data. Left column: Overall classification accuracy for different kernel types and numbers of principal components (PCs), with regularization parameter C = 1,10,100. For each kernel, the best performance was achieved using 2 or 3 PCs, as indicated on each bar. Middle column: PCA score plots showing separation along PC1 (98.06% of explained variance, top), PC2 (1.15%, middle), and PC3 (0.48%, bottom) corresponding to the components with the highest contribution to class separation. The species are represented by *L. amazonensis* (blue points) and *L. braziliensis* (red points). Right column: PCA loading plots showing the spectral regions (in cm^–1^) that most contribute to PC1, PC2, and PC3. Green curves represent the loading values, the green dashed horizontal line indicates the zero-reference baseline, and the gray dashed line represents the FTIR-SNV-FFT average spectra.

The optimum number of PCs was determined by retaining the lowest number that conveyed meaningful spectral information, as confirmed by loading plots, while excluding higher-order PCs dominated by noise. In standard practice, the selection is made by tracking LOOCV accuracy as a function of the number of PCs and stopping at the first maximum. In this data set, however, the first three PCs alone explained more than 99% of the variance, displayed clear species-level clustering, and achieved maximum accuracy in both LOOCV and external validation (Figure 5). Consequently, the analysis was limited to three PCs, as adding further components would only introduce noise without improving classification performance or generalizability.

The right column of Figure 5 displays the corresponding loading plots and clustering of the data in the PCA space (swarm plot). The loadings indicate that the first three principal components capture vibrational features relevant to class discrimination. While PC1 contributes most significantly to class separation, projections onto PC2 and PC3 also provide complementary information that enhances the robustness of SVM classification.

Finally, the predictive models were validated through a blind test using 30% of the samples that were not included in the training phase. Figure 6 summarizes the best-performing model, which corresponds to the Linear SVM using the first three principal components (PCs). This model demonstrated superior generalization capacity, as evidenced by its performance on the blind test. The small deviation observed between the leave-one-out cross-validation (LOOCV) and external validation results is not considered significant, likely due to sample size imbalance. In the blind test, the model achieved 100% accuracy, with both sensitivity and specificity also reaching 100%, indicating its robustness.

**Figure 6:**
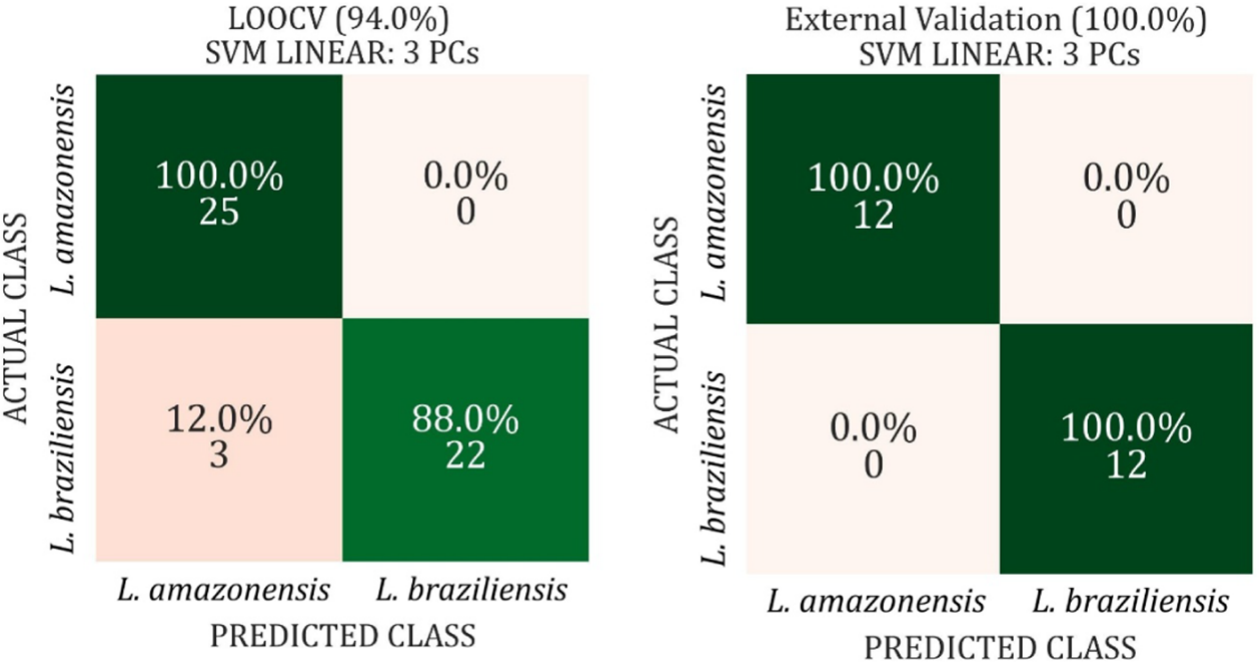
Confusion matrices illustrating the performance of the SVM classification model using three principal components (PCs) for cultured *Leishmania* promastigote samples. The left matrix shows the outcomes of leave-one-out cross-validation (LOOCV) on the remaining 70% of the data, while the right matrix presents the results of external validation using 30% of the data set (blind test). The input features were derived from PCA scores obtained from FTIR spectra preprocessed with Standard Normal Variate (SNV) and Fast Fourier Transform (FFT), within the spectral range of 1800–900 cm^–1^.

Notably, all tested kernel functions yielded models with overall accuracy above 90%, underscoring the potential of SVM-based approaches as reliable screening tools in microbiological laboratories. These models can serve as rapid and cost-effective alternatives to conventional gold standard methods. The primary advantages of the proposed approach include ease of implementation, minimal sample preparation requirements, and low operational cost, making it a promising strategy to accelerate diagnostic workflows across a variety of applications. Table 1, shows the performance metrics for external validation of the PCA–SVM model.

**Table 1:** Performance Metrics for External Validation (*n* = 24) of the PCA–SVM Model^a^

Class	Precision	Recall (Sensitivity)	F1-score	Support (n)	95% CI (Wilson)
*L. amazonensis*	100.0%	100.0%	100.0%	12	75.8%–100%
*L. braziliensis*	100.0%	100.0%	100.0%	12	75.8%–100%
Overall	100.0%	100.0%	100.0%	24	86.2%–100%

a Precision, recall (sensitivity), and F1-scores were 100% for both classes. Wilson 95% confidence intervals are reported to account for sample size uncertainty (12 samples per class).

Despite the promising results, this study should be interpreted as both robust and exploratory. While the spectral and computational approaches demonstrated high accuracy and generalization capacity, the data set was limited to two *Leishmania* species and a restricted number of cultured promastigote samples. To further validate and expand the applicability of this methodology, future studies should incorporate a broader diversity of *Leishmania* strains and clinical isolates from different geographic and epidemiological backgrounds. The approach can also be extended to include additional *Leishmania* species, potentially enabling the development of a comprehensive, FTIR-based classification system for the genus.

## 4 Conclusion

This study demonstrates that FTIR spectroscopy, coupled with supervised machine learning algorithms, enables accurate discrimination between *Leishmania amazonensis* and *Leishmania braziliensis* in axenic culture. The methodology proved to be fast, low-cost, and compatible with standard laboratory infrastructure, requiring minimal sample preparation—making it especially advantageous for resource-limited settings.

Beyond its immediate diagnostic potential, the approach represents a breakthrough in facilitating the work of clinical and research laboratories by offering a practical and scalable alternative to conventional molecular methods. The integration of spectral data analysis with AI provides a promising foundation for high-throughput parasite screening.

Despite the promising performance, the study should be viewed as a proof of concept. The current validation is limited to two species under controlled conditions. Therefore, future studies are essential to expand the data set, including a broader diversity of Leishmania species, strains, and clinical isolates from different geographical regions and host origins (human and animal). This will be critical to validate the method’s robustness and support its translation into field diagnostics.

Moreover, the proposed strategy aligns with One Health principles by providing a potential tool for integrated surveillance across human, animal, and environmental reservoirs. Its application could significantly enhance the monitoring and control of zoonotic transmission routes, contributing to more effective disease surveillance and management frameworks in endemic regions.
